# Pharmacists and pharmacy services in COVID-19 literature: A bibliometirc analysis

**DOI:** 10.1016/j.rcsop.2023.100243

**Published:** 2023-03-10

**Authors:** Abrar K. Thabit, Wajd S. Alsulmi, Nourah M. Aljereb, Omnia M. Khojah, Khadeja O. Almehdar, Manuel Jesús Cobo, Jimmy Jose, Antonio Vélez-Estévez

**Affiliations:** a Pharmacy Practice Department, Faculty of Pharmacy, King Abdulaziz University, Jeddah, Saudi Arabia; b Department of Computer Science and Artificial Intelligence, University of Granada, Granada, Spain; c School of Pharmacy, University of Nizwa, Nizwa, Sultanate of Oman; d Department of Computer Engineering, University of Cadiz, Spain

**Keywords:** Pharmacists, Pharmacy, COVID-19, Bibliometric analysis, COVID-19, Coronavirus disease of 2019, WHO, World Health Organization, DOI, Digital object identifier, SciMAT, Science Mapping Analysis Software Tool, MeSH, Medical subject heading

## Abstract

**Background:**

The COVID-19 pandemic had an enormous impact on the global economy and healthcare. Pharmacists were vital members of the healthcare system, and they participated in various strategies to reduce the effect of the pandemic. Numerous papers were published discussing their roles during the pandemic. Bibliometric analysis was used to measure the impact of publications on this topic and assessed them qualitatively and quantitatively over a specific time.

**Objective:**

Evaluate published literature pertaining to the roles of pharmacists and pharmacy services during the pandemic and identify gaps.

**Methods:**

An electronic search was conducted on PubMed database using a specific query. Eligible publications were published in English between January 2020 and January 2022 and discussed the role of pharmacists, pharmacies, and pharmacy departments during the pandemic. Clinical trials, studies on pharmacy education/training, and conference abstracts were excluded.

**Results:**

Of 954 records retrieved, 338 (35.4%) from 67 countries were included. Most papers (*n* = 113; 33.4%) were from the community pharmacy sector, followed by the clinical pharmacy sector (*n* = 89; 26.3%). Sixty-one (18%) papers were multinational, mostly involving two countries. The average number of citations of the included papers was 6 times (range 0–89). The most common MeSH terms were ‘humans’, ‘hospitals’, and ‘telemedicine’, where the former frequently co-appeared with the terms ‘COVID-19’ and ‘pharmacists.’

**Conclusions:**

Results from this study illustrate the innovative and proactive strategies developed by pharmacists during the pandemic. Pharmacists from around the world are encouraged to share their experiences for stronger healthcare systems to counter future pandemics and environmental disasters.

## 1 Introduction

The coronavirus disease of 2019 (COVID-19) has had an enormous toll on the healthcare and the economy across the globe since it was first identified in December 2019.^1^ Although the World Health Organization (WHO) did not recognize it as a pandemic until a few months into the crisis, healthcare systems and governments strived to contain the virus and minimize its spread. Healthcare professionals were hailed as heroes for their dedication and perseverance in delivering patient treatment.^2^

During the pandemic, pharmacists were vital members of the healthcare system. They were involved in institutional emergency preparedness in many hospitals, provided telemonitoring of inpatients, and participated in the development of global COVID-19 treatment and prevention guidelines.^3^ Moreover, community pharmacies delivered the medications to patients' homes to minimize contact. They also offered patient counseling and drug reconciliation using different interactive platforms.^4^ When COVID-19 vaccines became available, pharmacists worked as vaccination trainers, offered post-vaccination counseling, monitoring, and reporting of adverse events.^3^ Publicly, pharmacists had a significant role in the education about the disease, its symptoms, transmission, home remedies, and infection prevention. They also created awareness about the vaccines.^3^

Bibliometry is one branch of library and information science, whereas the term bibliometric analysis refers to the quantitative and qualitative analyses of bibliometric data reported in different kinds of publications on a certain topic.^5^ Such bibliometric data include the number of published articles, their themes, number of citations, top publishing journals, among other data. In other words, bibliometric analysis is a statistical tool used in various fields to measure the impact of publication on a particular topic or theme and assess it over a specific time.^6^ This method can highlight the most influential publications, journals, authors, countries, and organizations, and provide knowledge of the most current trends and research in several aspects that can be followed.^6^ Moreover, bibliometric analysis gives a reference to suggest numerous types of collaboration in the future among countries, institutions, and researchers.^7^

Numerous papers have been published from around the world discussing the crucial role pharmacists from various sectors played during the pandemic. However, based on the search conducted we could not find any bibliometric analysis published to date evaluating publications on the role of pharmacists and pharmacies in the pandemic. In relation to COVID-19 literature, bibliometric analysis will give an opportunity to understand what has been achieved and what needs to be achieved in light of the pandemic.^8^ Therefore, this study aimed to evaluate literature concerning the role of pharmacists and pharmacies in this pandemic and identify the gaps that guide researchers to conduct valuable studies by using bibliometric and visualization methods.

## 2 Methods

### 2.1 Study design and eligibility

This was a bibliometric analysis of studies describing the role of pharmacists, pharmacies, and pharmacy departments during COVID-19 pandemic that were published in English between January 2020 and January 2022. Prospective and retrospective clinical studies/trials, studies related to education and training in pharmacy, and conference abstracts were excluded. PubMed database was used for literature search. Duplicate records were identified through the titles and the digital object identifier (DOI), and were eliminated. This was followed by manual filtration of the retrieved unduplicated records for their eligibility by evaluating each article's abstract and/or full text.

While we acknowledge the fact that many of the published clinical studies involved pharmacists as part of the research teams,^3^ these studies were excluded as they involved clinical evaluation of patients data and the outcomes of different treatments; hence, they were deemed different from the articles that evaluated interventions and contributions made by pharmacists, and discussed the various roles they played during the pandemic. Therefore, such clinical studies were considered outside the scope of the current bibliometric analysis. To confirm that at least one of the authors of the eligible articles was a pharmacist, we evaluated the credentials and affiliations of all the authors, as well as their profiles on their workplace websites (e.g., university page) and reliable professional websites, such as LinkedIn and Research Gate.

### 2.2 Search query

The following string was used for the literature search:

TS = ((“Wuhan coronavirus” OR “Wuhan seafood market pneumonia virus” OR “Covid19*” OR “Covid-19*” OR “Covid-2019*” OR “coronavirus disease 2019” OR “SARS-CoV-2” OR “sars2” OR “2019-nCoV” OR “2019 novel coronavirus” OR “severe acute respiratory syndrome coronavirus 2” OR “2019 novel coronavirus infection” OR “coronavirus disease-19” OR “novel coronavirus” OR “coronavirus” OR “SARS-CoV-2019” OR “SARS-CoV-19” OR “coronavirus” OR “coronavirus disease” OR “) AND (Pharmacy OR Pharmacist).

### 2.3 Bibliometric analysis

The bibliometric analysis was based on co-words networks and science mapping analysis. To that end, Science Mapping Analysis Software Tool (SciMAT; version 1.1.04, University of Granada, Spain) was employed due to its preprocessing capabilities, visualization techniques, and strong methodology.^9^ It should be highlighted that SciMAT was designed according to the science mapping analysis approach presented, combining both performance analysis tools and science mapping tools to analyze a research field and detect and visualize its conceptual subdomains (particular topics/themes or general thematic areas) and its thematic evolution.^10^

The science mapping analysis took place in three stages: 1) Research themes detection. In the period of time studied (i.e., two years, from January 2020 to January 2022), the corresponding research themes were identified by applying a co-word analysis to the raw data of all the published papers in the research field. This was followed by keywords clustering to topics/themes that located keyword networks strongly linked to each other and that correspond to centers of interest or to research problems that were of significant interest among researchers. The similarity between the keywords was evaluated using the equivalence index. 2) Visualizing research themes and thematic network. In this phase, the detected themes were visualized by two different visualization instruments: strategic diagram and thematic network. Each theme was characterized by two measures: centrality and density. Centrality measures the degree of interaction of a network with other networks, whereas density measures the internal strength of the network. Using both measures, a research field could be visualized as a set of research themes, mapped in a two-dimensional strategic diagram and classified into four groups: motors (upper-right), well developed and isolated (upper-left), weakly developed and marginal (lower-left), and basic and transversal (lower-right). 3) Performance analysis. In this stage, the relative contribution of the research themes to the whole research field was measured (qualitatively and quantitatively) and used to establish the most prominent, productive and highest-impact subfields. Some of the bibliometric indicators included the number of published documents and number of citations.^11^ For each theme, the performance measures were computed considering the documents associated with it.

## 3 Results

**Fig. 1:**
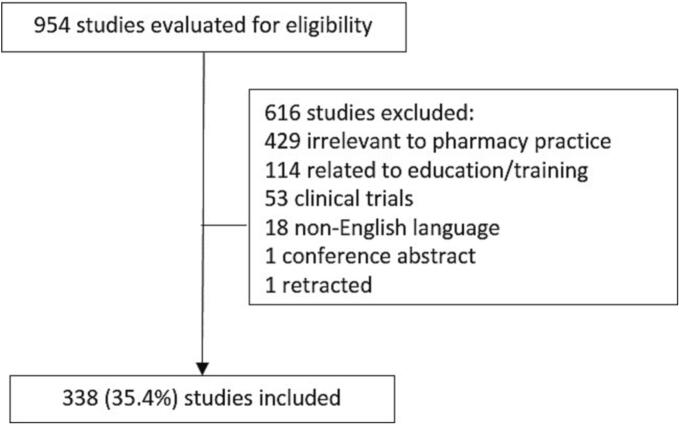
Flowchart of included studies.

A total of 954 studies were retrieved after duplicate removal. Following manual filtration, 338 (35.4%) from 67 different countries met the eligibility criteria and were included. Fig. 1 shows the flowchart of the publications and the reasons for their exclusion. Among the included publications, 135 (39.9%) were published in 2020, while 203 (60.1%) were published in 2021. Most of the included articles (*n* = 324; 95.6%) had at least one pharmacist among the authors. The remaining 14 articles were authored by either medical writers or other healthcare providers who commented on the role the pharmacists played in certain interventions made during the pandemic, such as telehealth services, medication delivery, and vaccine administration.

**Fig. 2:**
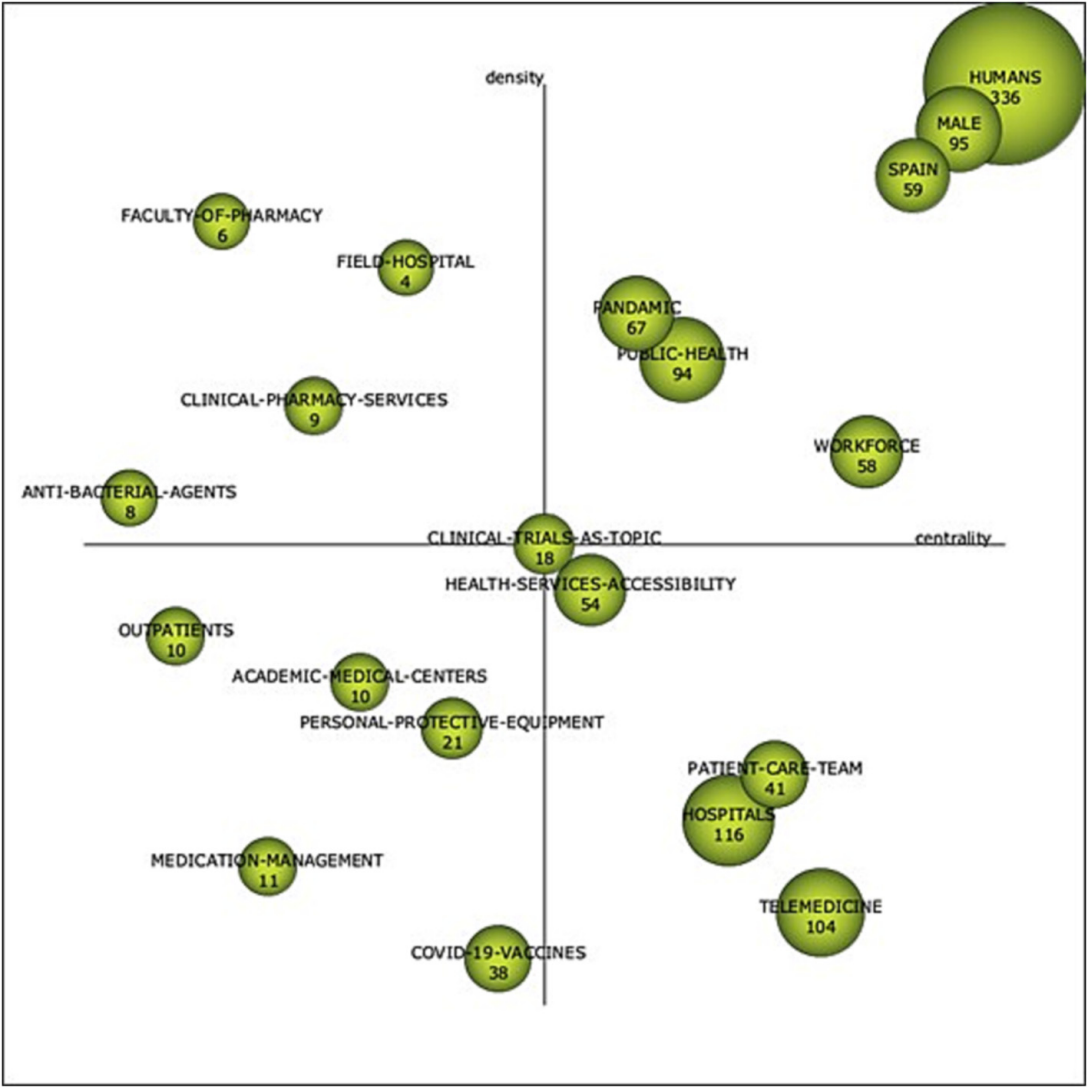
Map of the most common MeSH terms in the included publications.

**Fig. 3:**
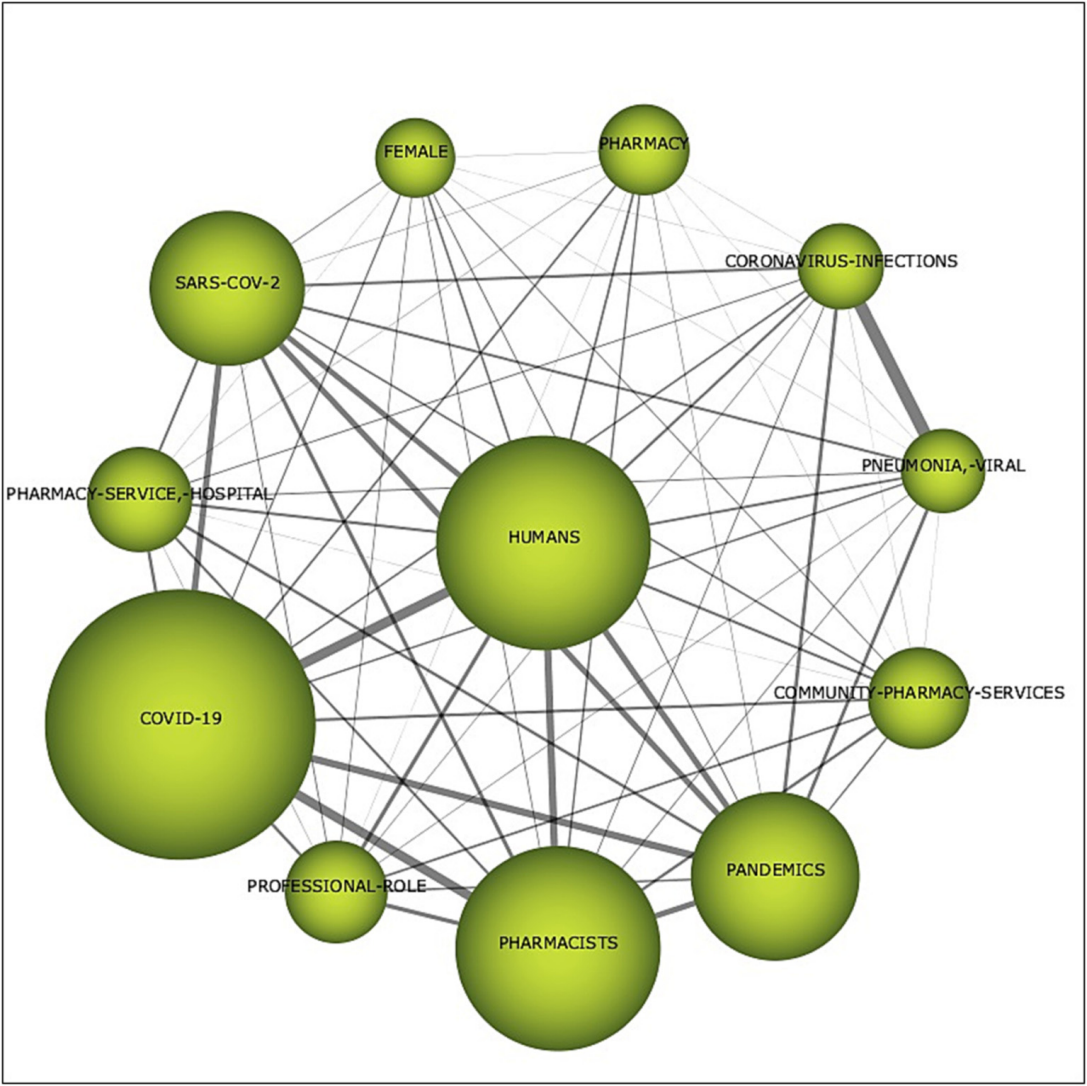
The co-word network of the MeSH term ‘humans’.

Table 1 summarizes the pharmacy areas and foci of the publications included. Most papers 113 (33.4%) were from the community pharmacy sector, followed by the clinical pharmacy sector with 89 (26.3%) papers. From the focus perspective, 129 (38.2%) of the publications had multiple focuses between administration, COVID-19 treatment, prevention, and public education. A map of the most frequently used medical subject heading (MeSH) terms in the included publications is shown in Fig. 2, where the MeSH term ‘humans’ was the most reported, followed by ‘hospitals’, ‘telemedicine’, and ‘public health.’ Each of the terms reported in Fig. 2 is the representative term of a group of MeSH terms underneath them that were very frequently co-appeared with them. For instance, the term ‘humans’ was the most central term in its subnetwork of closely related terms that included ‘COVID-19’ and ‘pharmacists’ (Fig. 3).

**Table 1:** Pharmacy areas and focuses of reported studies (*n* = 338).

Parameter	N (%)
Area • Community pharmacy⁎ • Clinical pharmacy⁎⁎ • Hospital pharmacy⁎⁎⁎ • More than one sector	113 (33.4%) 89 (26.3%) 55 (16.3%) 81 (24%)
Focus • Administrative • Treatment • Prevention • Public education • More than one focus	78 (23.1%) 65 (19.2%) 56 (16.6%) 10 (2.9%) 129 (38.2%)

⁎ Defined as the practice of processing and verifying prescriptions followed by preparation, dispensing, and patient counseling in retail pharmacies (i.e., embedded within the community). Community pharmacists are considered primary healthcare providers as they are the most accessible healthcare professional to the public.^14^

⁎⁎ Defined as the provision of pharmacotherapeutic management in direct patient care settings (such as hospital wards and outpatient clinics) by optimizing medication therapy either independently or in collaboration with other healthcare professionals to ensure appropriate, safe, and cost-effective use of medications.^15^

⁎⁎⁎ Defined as the practice of processing and verifying medication orders (for inpatients) or prescriptions (for outpatients) followed by preparation and dispensing within the hospital. Hospital pharmacy practice also involves sterile and non-sterile compounding of medications.^14^

**Fig. 4:**
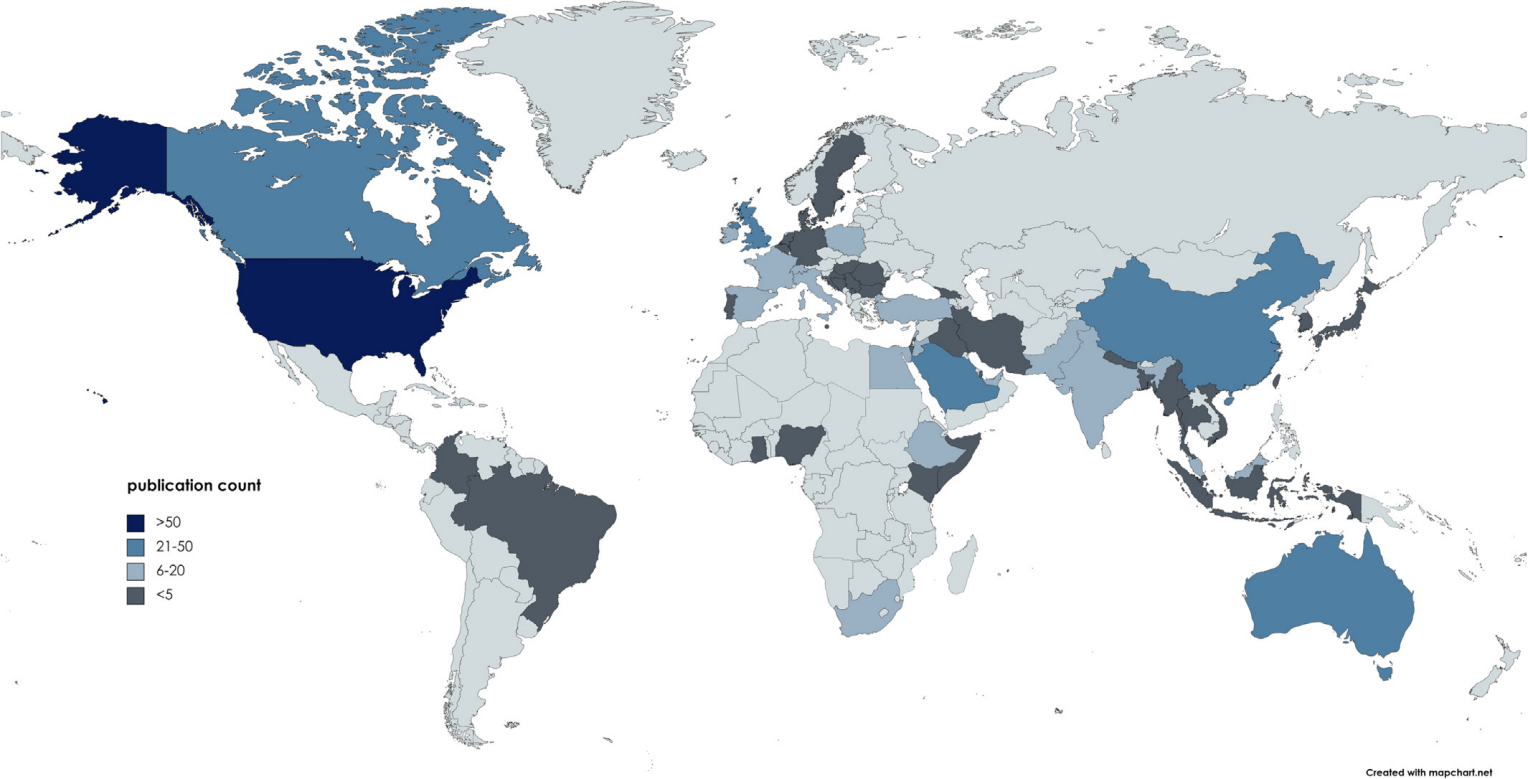
Distribution of the countries with the highest publications about the role of pharmacists during COVID-19.

The included publications came from 67 different countries (34.3% of 195 countries in the world). Most publications came from the United States (*n* = 106; 31.3%), followed by the United Kingdom (*n* = 25; 7.3%). From the Middle East, Saudi Arabia contributed the largest with 23 (6.8%) publications, followed by Jordan with 18 (5.3%). Fig. 4 depicts the distribution of countries from which the included publications were produced. Moreover, about one-fifth (*n* = 61; 18%) of the published papers involved collaborators from different countries, mostly involving two countries and the largest collaboration involved 12 countries (Table 2). The country that was mostly involved in multinational works was the United Kingdom, followed by the United States and Australia (Table 3).

**Table 2:** Characteristics of included studies (*n* = 338).

Characteristic	N (%)
Multinational studies	61 (18%)
Number of citations	6 (0–89)⁎
Top journals (published ≥7 papers)	
• *Research in Social & Administrative Pharmacy*	45 (13.3%)
• *American Journal of Health-System Pharmacy*	42 (12.4%)
• *Journal of the American College of Clinical Pharmacy*	17 (5%)
• *Journal of the American Pharmacists Association*	16 (4.7%)
• *Farmacia Hospitalaria*	11 (3.2%)
• *Journal of Pharmaceutical Policy and Practice*	9 (2.6%)
• *Pharmacy*	9 (2.6%)
• *International Journal of Environmental Research and Public Health*	7 (2%)
• *Journal of Pharmacy Practice*	7 (2%)

⁎ mean (range).

**Table 3:** Data of multinational studies (*n* = 61).

Parameter	N (%)
Number of countries • 2 • 3 • 4 • ≥ 5	32 (52.4%) 14 (23%) 7 (11.5%) 8 (13.1%)
Most common countries involved • United Kingdom • United States • Australia	22 (36%) 17 (27.8%) 16 (26.2%)

As shown in Table 2, the journal *Research in Social & Administrative Pharmacy* published the majority of the included studies (*n* = 45; 13.3%) followed by the *American Journal of Health-System Pharmacy* with 42 (12.4%) papers. In general, the papers included in this analysis were cited at an average of 6 ± 12. The top three cited papers were “On the frontline against COVID-19: Community pharmacists' contribution during a public health crisis” by Cadogan et al. (2021), “Recommendations and guidance for providing pharmaceutical care services during COVID-19 pandemic: A China perspective” by Zheng et al. (2021), and “Community pharmacist in public health emergencies: Quick to action against the coronavirus 2019-nCoV outbreak” by Ung et al. (2020), which were cited 89, 82, and 75 times, respectively. The five most commonly cited publications and their details are listed in Table 4.

**Table 4:** Details of the five top cited articles.

Authors	Article Title	Journal	Document Type	Publication Year	Volume	Issue	Pages	Times Cited
Cadogan et al	On the frontline against COVID-19: Community pharmacists' contribution during a public health crisis	*Research in Social & Administrative Pharmacy*	Review	2021	17	1	2032–2035	89
Zheng et al	Recommendations and guidance for providing pharmaceutical care services during COVID-19 pandemic: A China perspective	*Research in Social & Administrative Pharmacy*	Original article	2021	17	1	1819–1824	82
Ung et al	Community pharmacist in public health emergencies: Quick to action against the coronavirus 2019-nCoV outbreak	*Research in Social & Administrative Pharmacy*	Original article	2020	16	4	583–586	75
Liu et al	Providing pharmacy services during the coronavirus pandemic	*International Journal of Clinical Pharmacy*	Review	2020	42	2	299–304	62
Erku et al	When fear and misinformation go viral: Pharmacists' role in deterring medication misinformation during the ‘infodemic’ surrounding COVID-19	*Research in Social & Administrative Pharmacy*	Review	2021	17	1	1954–1963	59

## 4 Discussion

To our knowledge, this is the first bibliometric analysis of publications discussing the role of pharmacists, pharmacies, and pharmacy departments during the pandemic. Healthcare providers suffered from stress and burnout and had to augment their efforts to adapt with the challenges imposed by COVID-19 on healthcare and public health.^7^ As vital members of the healthcare system, pharmacists contributed in various ways to reduce the effect of the pandemic. They have also participated in educating the public about risks of the infection and how to prevent acquiring it. One of the major contributions pharmacists made in support of their global peers was sharing their experiences and the innovative strategies they carried out in the different sectors in which they work. Such contributions helped pharmacists from around the world learn about what worked and what did not; hence, selecting the most successful interventions and applying them to their area of practice.

Several articles were published on the role of pharmacists and pharmacies during the pandemic through the peak period after January 2020. Most of the articles were published in 2021 compared with the number of articles published in 2020. This observation was expected because more data were available over time, which allowed time for strategies to be implemented and evaluated. Furthermore, it was also expected that at least one pharmacist was among the authors in the majority of the included articles (95.6%). The 14 articles that did not include a pharmacist author either described interventions that were usually multidisciplinary involving different healthcare providers, such as physicians or nurses describing telehealth services, or were written by a medical writer (i.e., journalist) for a pharmacy journal who highlighted the roles of the pharmacists during the pandemic from different aspects.

Since pharmacy is a multidisciplinary specialty, pharmacists from different sectors contributed to the literature with their experiences. The majority of the papers discussed the community pharmacy sector accounting for 113 (33.4%) of the publications. This in part could be due to the fact that community pharmacy is the major sector pharmacists from around the world work at. A second potential explanation is that community pharmacists represent the first contact with patients and the community, where they play an important role in providing direct patient care and counseling. While hospitals and health institutions restricted access during the pandemic, patients found community pharmacies an easy and accessible provider to meet their healthcare needs. Pharmacists' roles in addressing the pandemic included treatment, preventing the spread of the infection, healthcare system management and administration, and public education. Interestingly, the greatest number of studies dealt with administrative aspects. This could be explained by the unique nature of the situation, where pharmacists or pharmacies faced various administrative challenges during this period, like any other profession during the pandemic.

While pharmacists from >67 countries participated in literature enrichment with their experiences during the pandemic, pharmacists from the United States were the top contributors. This could be due to the advanced pharmacy services offered there, the large population of pharmacists, and the fact that it is the leading country in scientific publications.^12^ Nonetheless, other countries have also contributed to the literature and shared their innovative strategies in dealing with the pandemic, such as the United Kingdom with 25 (7.3%) publications. From the Middle East, Saudi Arabia and Jordan contributed the most with 23 (6.8%) and 18 (5.3%) papers, respectively, which reflect their concerns about public health and sharing their experiences with their fellow pharmacists. Notably, 61 (18%) of the publications were multinational showing the collaborative efforts of pharmacists from different countries. In general, it is noted that multinational or collaborative research made a rapid increase during the pandemic considering it was a global issue where in contributions from different parts of the world became essential and collaboration turned out to be the approach in practice. Further, the increased interest, ease, and practice of getting in touch with researchers from around the world through online modes during the pandemic might have facilitated collaborative research.

The top journals were *Research in Social & Administrative Pharmacy* (RSAP) and the *American Journal of Health-System Pharmacy* (AJHP), which have impact factors of 3.348 and 2.980, respectively. The aim and scope of RSAP pertains to pharmacy administration, whereas AJHP is more of a multidisciplinary journal. In addition to being indexed in PubMed, all four top journals (including *Journal of the American College of Clinical Pharmacy* and *Journal of the American Pharmacists Association*) are also indexed in the Institute of Science Information/Web of Science database, which indicates that such journals are high quality, well-established in their fields, and their published papers have undergone rigorous peer-review.

This is the first study to evaluate the literature concerning the roles of pharmacists during the pandemic; nevertheless, it was limited by including only PubMed database. PubMed was selected because it indexes >30,000 journals, including MEDLINE and non-MEDLINE journals.^13^ Additionally, more than half of the retrieved articles (*n* = 616; 64.6%) were irrelevant to the topic of the study; hence excluded. This could be possibly due to algorithmic issues of the software. Lastly, this was a bibliometric analysis of the literature where it only evaluated quantity and the bibliographic data of articles published on the roles of pharmacists and pharmacies during the pandemic. It should be noted that a bibliometric analysis is different from narrative or systematic reviews, which evaluate and summarize the content of the retrieved articles.^5^ As such, a detailed literature review of the retrieved records in the current study and a summarization of their findings could have provided a better insight into their content; though, this was not within the scope of this study.

## 5 Conclusion

This bibliometric analysis demonstrated that pharmacists were proactive in contributing to the literature during the pandemic by sharing their experiences, which is beneficial for their fellow pharmacists to help improve and enhance pharmacy services. Pharmacists from around the world, especially countries with no or low publication records, are encouraged to share their experiences to help create stronger healthcare systems to counter future pandemics and environmental disasters.

## Funding

None.

## Declaration of competing interest

The authors declare no conflict of interest.

## Data Availability

Data associated with this study are available on Open Science Framework at: https://osf.io/w2kga/?view_only=1af6a528fbfc4ef98e722b4ad8084b72.
